# On the use of structured light in nonlinear optics studies of the symmetry group of a crystal

**DOI:** 10.1038/srep20906

**Published:** 2016-02-08

**Authors:** Rocio Jáuregui, Juan P. Torres

**Affiliations:** 1 Instituto de Física, Universidad Nacional Autónoma de México, Apartado Postal 20-364, 01000 México D.F., México; 2 ICFO–Institute of Photonic Sciences, The Barcelona Institute of Science and Technology, Mediterranean Technology Park, 08860, Castelldefels, Barcelona, Spain; 3 Dep. Signal Theory and Communications, Universitat Politecnica de Catalunya, Jordi Girona 1-3, 08034 Barcelona, Spain

## Abstract

We put forward a technique that allows to extract information about the symmetry group to which certain nonlinear crystals belong using a *single* illuminating beam. It provides such information by considering the outcome of a nonlinear optics process characterized by the electric nonlinear susceptibility tensor, whose structure is dictated by such symmetry group. As an example, we consider the process of spontaneous parametric down-conversion, when it is pumped with a special type of Bessel beam. The observation of the spatial angular dependence of the lower-frequency generated light provides direct information about the symmetry group of the crystal. We should stress that the choice of the appropriate illumination is of paramount importance for unveiling the sought-after information.

The spatial arrangements of atoms of substances that can present themselves in a crystalline structure is determined by the specific (*point group* or *symmetry group*) category^1^ to which the crystal belongs. According to Neumann’s principle^2^, or principle of symmetry, if a crystal is invariant with respect to a set of symmetry transformations, any physical property of the crystal is also invariant under such operations. Thus, the response of a crystal to optical, electric or magnetic stimulus can be used to predict general features of its structure, i.e., its symmetry group, allowing to unveil its presence in a determined spatial region of interest.

Crystallographic characterization is usually performed by diffraction techniques where X-rays^3^, or material waves^4^,^5^,^6^ interact with the atoms that are arranged in a certain pattern dictated by the symmetry group. By observing the spatial distribution of the photons or particles that come out from the crystal, one can obtain the sought-after information. In spite of the high importance of such techniques, sometimes they cannot be used, or its use is cumbersome and extremely difficult. Moreover, they are not immune to obstacles that may prevent a unique symmetry assignment to a given diffraction pattern. This can be due, e. g., to insufficient number of Bragg peaks owing to a finite instrument momentum range or spurious peaks arising from multiple scattering events. In these cases, a companion technique might help to identify the appropriate symmetry group of the crystal.

An alternative to diffraction techniques is the characterization of the crystal symmetry by its effects on the nonlinear optical response encoded in the susceptibility tensors^7^,^8^,^9^. These tensors are particularly useful because of their high sensitivity to both lattice and electronic symmetries. From elementary electric transitions of the components of a material, the companion electric polarization induced in it, when it is illuminated by an optical beam, can be written as the Bloembergen expansion^10^


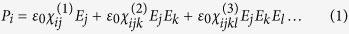


where *E*_*i*_ (*i* = *x*, *y*, *z*) are the components of the electric field, 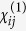 is the linear susceptibility tensor that determines the linear polarization response of the material, and 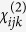, 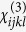 … are nonlinear susceptibility tensors of different ranks responsible for the nonlinear polarization generated in the medium.

Here we show two main things. Firstly, that it is possible to perform complementary symmetry studies of nonlinear materials by using nonlinear optics processes besides second harmonic generation (SHG), such as spontaneous parametric down-conversion (SPDC). In SPDC, an intense pump beam with frequency *ω*_*p*_ interacts with the atoms or molecules of a second order nonlinear crystal with nonlinear coefficient 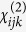. In the process, a flow of paired photons is generated, the signal and idler, with central frequencies *ω*_*s*_ and *ω*_*i*_, such that *ω*_*s*_ + *ω*_*i*_ = *ω*_*p*_.

Secondly, that instead of illuminating the nonlinear crystal with paraxial Gaussian beams that propagate along a myriad of different directions, as it is usually done in similar cases^8^, it is more advantageous to choose as illuminating beam a non-paraxial optical beam. The use of a laser source with fast switching of its pointing direction might be technically cumbersome, and can severely limit the applicability of the method due to the need to control mechanical noise. However, the use of an appropriately designed non-paraxial beam allows to unveil critical information on the crystal symmetry group in a experiment using a single structured pump beam.

## SPDC using structured pump beams designed for crystallographic studies

Under standard conditions^11^, most SPDC configurations consist of a Gaussian pump beam that impinges normally onto the surface of the nonlinear crystal, which is endowed with a second order nonlinear electric susceptibility 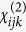. The cut angle of the crystal is chosen to guarantee the fulfillment of the phase matching conditions, which are equivalent to the conservation of energy and momentum of the photons involved in the SPDC process. In most cases, the pump beam is linearly polarized. Under these circumstances, the distribution of wave vectors of the signal and idler photons, for given frequencies of the resulting photon pairs, is restricted to a single cone (in the case of type I SPDC) or to two cones (in the case of type II SPDC) with axes symmetrically arranged relative to the pump beam^12^. These conical distributions are observed for different crystal symmetries, so they do not bear useful information about the point group to which the crystal belongs. This is because in the configuration considered, the nonlinear process addresses only certain elements of the nonlinear susceptibility tensor.

We show that a different configuration can lead to a spatial distribution of the photon pairs that directly reflects the symmetry group of the crystal. For the sake of simplicity, we will consider the case of an uniaxial birefringent crystal with its optic axis parallel to the normal of its surface. The pump beam that illuminates the crystal is a vectorial Bessel mode^13^,^14^, prepared to guarantee a vectorial extraordinary character inside the nonlinear media. This can be done by choosing a transverse magnetic (TM) mode with its main propagation direction coincident with the axis of the nonlinear crystal.

**Figure 1:**
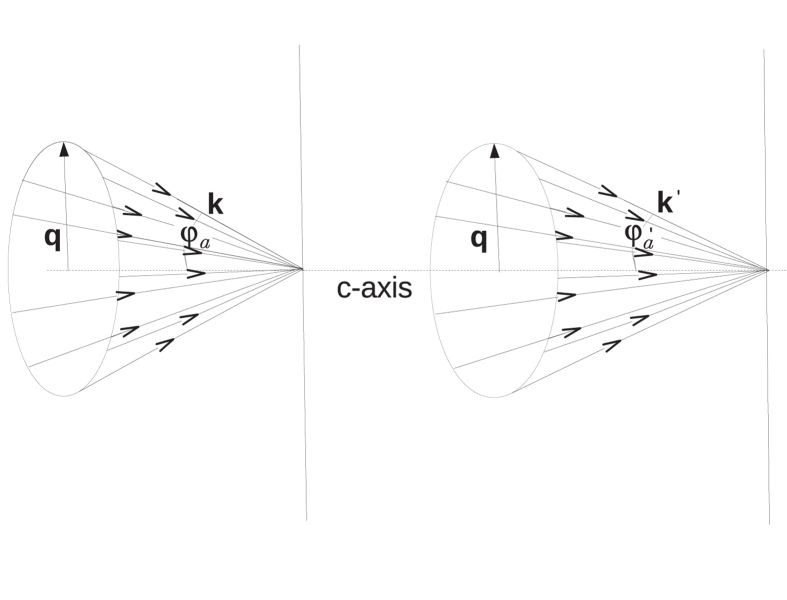
A Bessel beam is formed by a conical superposition of plane waves each one with a wavevector **k**. The aperture of the cone is denoted by *φ*_*a*_. Inside the uniaxial birefringent crystal with an optical **c**-axis–parallel to the main direction of propagation of the beam– it is refracted and the aperture of the cone is modified to 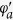. The geometry of the set up guarantees that the transverse component of each plane wave in the superposition–here denoted generically by **q**– is not modified, while the component parallel to the **c**-axis, satisfies the dispersion relations given in Eq. (5) depending on the ordinary or extraordinary character of the wave.

A Bessel beam is formed by the superposition of plane waves with wave vectors confined in a cone and with a circular cylindrical symmetry on the angular spectrum. In this way, the cylindrical symmetry of the photon pairs produced in the SPDC process is directly broken by the intrinsic symmetry of the crystal, which manifests on the spatial distribution of the resulting photon pairs. The geometry of the set-up is illustrated in Fig. 1. The axicon angle *φ*_*a*_ of the incident vectorial Bessel mode is the parameter to be optimized for both the fulfilment of the phase matching conditions and to obtain a clear visibility of the characteristic crystallographic pattern built by the photon pairs.

Note that choosing a Bessel beam as a pump of the nonlinear process is equivalent to observing the crystal structure simultaneously for many different angles. In this configuration, SPDC (or any other nonlinear optical process) is sensible to all the components of the nonlinear optical susceptibility tensor, including that along the main direction of propagation of the pump beam; that is due to the fact that TM modes acquire a significant component of its electric field along their main direction of propagation as they depart from the paraxial limit^14^.

In this paper we consider vectorial monochromatic beams, i.e., beams with an electric field 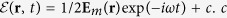 where *ω* is the angular frequency of the beam, **r** designates the spatial location, *t* is time, **E**_*m*_ is the vectorial spatially-varying amplitude of the beam and *m* is related to the orbital angular momentum of the beam.

In free space, the electric field of a transverse-magnetic (TM) Bessel beam of order *m*, which is an exact solution of Maxwell’s equations, can be written^13^ as a sum of plane-waves with equal amplitude and wavevector confined in a cone around the **z**-axis, the main direction of propagation of the beam, with axicon angle *φ*_*a*_, i.e.,


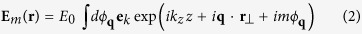


where 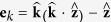 is the polarization of each plane-wave with wavevector **k**; this vector can be written as a sum of a longitudinal vector with amplitude 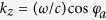, and a transverse wavevector 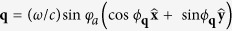; *ϕ*_**q**_ is the polar angle around the **z**-axis of each wavevector **k**, it can take any value between 0 and 2*π*, with zero giving the orientation of the 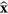-axis. Notice that even though the polarization of each **k**-wave is perpendicular to **k**^15^, the superposition of all **k**-waves yields a beam with a non-zero field component along the main direction of propagation 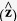. For paraxial beams (*φ*_*a*_ small) and *m* = 0, one obtains the so-called radial modes^13^,^16^.

In experiments, one never generates such an ideal beam. However, one can produce vectorial Bessel beams close to the one described by Eq. (2) by superposing scalar Bessel modes with the proper topological charge *m* and polarization^17^. Scalar Bessel modes were generated for the first time by illuminating, with a quasi-plane wave, an annular slit placed in the back focal plane of a lens^18^, later an alternative to this method was found on the usage of a lens with a conical surface –called an axicon– with cone angle *φ*_*α*_ 19; other options for the generation of Bessel beams are implemented using a space light modulator, or a structured diffraction plate. In most circumstances, the generated beam is designed to be close to the paraxial limit, i. e. 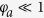, even though there is no fundamental limitation on achieving higher values of *φ*_*a*_. Under experimental conditions, the wavevectors **k** are no longer confined to a cone of angle *φ*_*a*_, with transverse wavevector **q**, but instead the wavevectors spread narrowly around a central value, **q** + Δ**q**, or equivalently, *φ*_*a*_ + Δ*φ*_*a*_, with 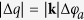.

The Hamiltonian of interaction of SPDC is^20^


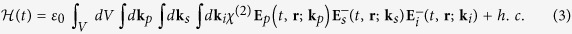


where *V* is the volume of interaction and 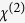 is the nonlinear tensor that characterizes the nonlinear electric response of the material.

Let us consider as example a type I (*eoo*) SPDC process in an uniaxial crystal^21^. A configuration that will give us information about the symmetry of the crystal is the one where the pump beam propagates inside the nonlinear crystal along the optic axis 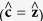. The signal and idler waves propagate as ordinary waves. For the case of an intense classical extraordinary pump beam that generates ordinary signal and idler photons, the pump beam, and the electric field operators for signal and idler photons write^11^


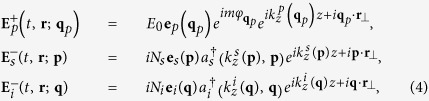


where *N*_*s*_ and *N*_*i*_ are normalization factors,


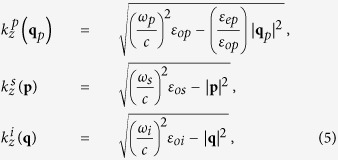


*ε*_*op*,*os*,*oi*_ are the ordinary relative permittivities inside the crystal of the pump, signal and idler waves respectively, *ε*_*ep*,*es*,*ei*_ are the corresponding extraordinary ones, and


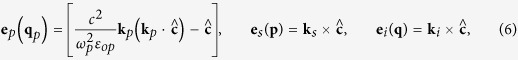



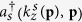
 and 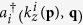 are creation operators for signal and idler photons, with momentum 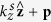 and 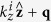, respectively.

Since in most situations the nonlinear interaction is weak, we can obtain an accurate quantum description by calculating the first-order solution of the Schrödinger equation, i.e.,


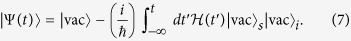


The interaction Hamiltonian is effectively zero when the classical beams that pump the nonlinear process are zero, so that, the time of integration can be extended to *t* = ∞. The quantum state of the down-converted photons at the output face of the nonlinear crystal, neglecting for the sake of simplicity the contribution from the vacuum term, can be written as


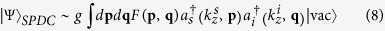


where


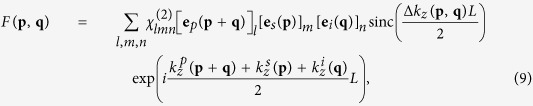



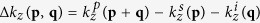
, and 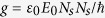 encompasses the amplitudes of the electric fields of the pump beam, and of the signal and idler photons.

It is important to stress out the crucial role that the tensorial character of 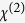 plays in the SPDC configuration considered. Most experiments that make use of SPDC, due to the paraxial character of the pump, can be described using an effective nonlinear index^21^ that writes 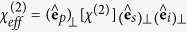, where 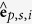 are the linear polarizations of the pump, idler and signal photons, respectively. The flux of down-converted photons depends on the magnitude of the effective index. The vectorial structure of the electromagnetic field defines its polarization and, consequently, affects its total angular momentum. Thus, non paraxial pump beams are expected to yield interesting results determined by the structure of 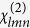 and its relation to the angular momentum content of the down-converted photons^22^.


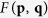
 describes an entangled state in the idler and signal degrees of freedom. The flux rate of detections of a signal photon with transverse wavevector **p** in coincidence with an idler photon with transverse wavevector **q** (*coincidence detection*), with resolutions Δ**p** and Δ**q** respectively, is


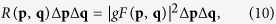


while the flux rate of signal photons detected with transverse wavevector **p** and resolution Δ**p** (*singles detection*) writes


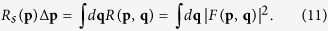


**Figure 2:**
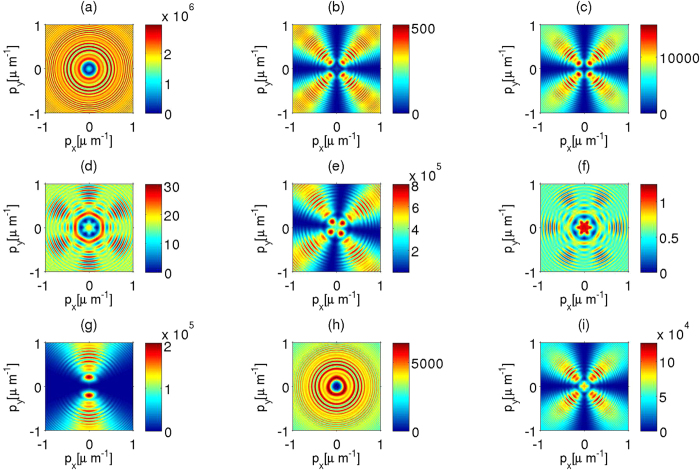
Angular dependence of the flux rate 
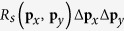
 of signal photons detected [*singles detection* given by Eq. (11)] for type I degenerate SPDC (*λ*_*s*_ = *λ*_*i*_ = 2*λ*_*p*_). (**a**) 2-Furyl Methacrylic Anhydride (FMA): point group 4 mm (C_4v_); (**b**) KH_2_PO_4_ (KDP): point group 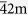 (*D*_2*d*_); (**c**) CsH_2_AsO_4_: point group 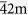 (*D*_2*d*_); (**d**) GaSe: point group 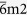 (D_3*h*_); (**e**) HgGa_2_S_4_: point group 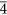 (S_4_); (**f**) HgS: point group 32 (D_3_); (**g**) LiNbO_3_: point group 3 m (C_2v_); (**h**) *β*-BaB_2_O_4_ (BBO) point group 3 m (C_3v_); (**i**) KD_2_PO_4_ (DKDP) point group 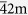 (D_2*d*_). The components of the tensor 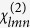 are obtained from the data given in ref. 21, and using Kleinman symmetry relations. The wavelength of the incident TM Bessel mode is *λ*_*p*_ = 407 nm; the reported values correspond to the expected photon counts per second that would be obtained with a resolution detection of 0.005 *μ*m^−1^ and a pump beam of 30 mW. The angle of the cone that characterizes the illuminating pump Bessel beam is sin*φ*_*a*_ = 0.097. We also consider a spread of directions Δ*φ*_*a*_ ~ 0.0004 rad. The crystal length is taken as *L* = 1 mm.

Let us illustrate these results and how they help to determine the symmetry group of the crystal. In Fig. 2, we exemplify the angular dependence of the flux rate of signal photons (singles detections as given by Eq. (11)) for a variety of nonlinear crystals with different crystallographic symmetries. The numerical simulations consider the adequate Sellmeier equations, the crystal nonlinear properties as given in ref. 21, high resolution of **p** (0.005 *μ*m^−1^), and a moderate value of its intensity (30 mW). For small values of the transverse wavevector of a *λ*_*p*_ = 407 nm pump beam (
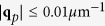
 which corresponds to an angle 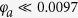) the angular spectrum is always formed by a set of concentric rings similar to those observed in Fig. 2(a). As 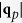 increases the down-converted photons are emitted in a wider region in the transverse wavevector space, and the symmetry of the crystal becomes more evident; so that the crystallographic structure is directly observable for 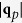 values above a crystal dependent threshold. In Fig. 2, that threshold has already been exceeded at **q**_*p*_ = 0.15 *μ*m^−1^ for GaSe, and crystallographic traces are evident in its angular spectra. GaSe is a layered crystal that belongs to the space group D_3*h*_; an individual layer consists of four planes of Se − Ga − Ga − Se with the Ga − Ga bond normal to the layer plane arranged on a hexagonal lattice, and the Se anions located in the eclipsed conformation when viewed along the optic axis^9^; these leads to the hexagonal shape of the corresponding maximum flux rate regions. A key point concerning the determination of the symmetry group of a crystal comes from the fact that structurally characteristic patterns on the flux rates *R*_*s*_(**p**) arise from observations with a common moderate axicon angle as illustrated in Fig. 2. As a consequence a direct comparison between numerical simulations for moderate – though, in general, out of the paraxial regime−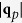 values and the observed results would yield the desired identification of the symmetry group. This comparison can be automatized in actual experimental set ups. Properties such as the radius of the concentric rings, the values of 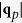 at which the visibility of the symmetry of the crystal is significant, and the maximum value of *R*_*s*_, could be used to determine some characteristics of the linear susceptibility tensor and the second- order nonlinear susceptibility tensors. For instance, KDP (Fig. 2(b)), CsH_2_AsO_4_ (Fig. 2(c)) and DKDP (Fig. 2(i)) belong to the same point group, 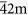, but they have slightly different values of *ε*_*o*_, *ε*_*e*_ and the relevant components of 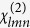. This manifests in general common properties between the angular spectrum of those crystals, but there are still measurable differences of the relative values of *R*_*s*_ for each one of the crystals, for small values of **p**. These differences are mainly due to a higher sensitivity of CsH_2_AsO_4_ to the electric field component along the **z**-axis.

**Figure 3:**
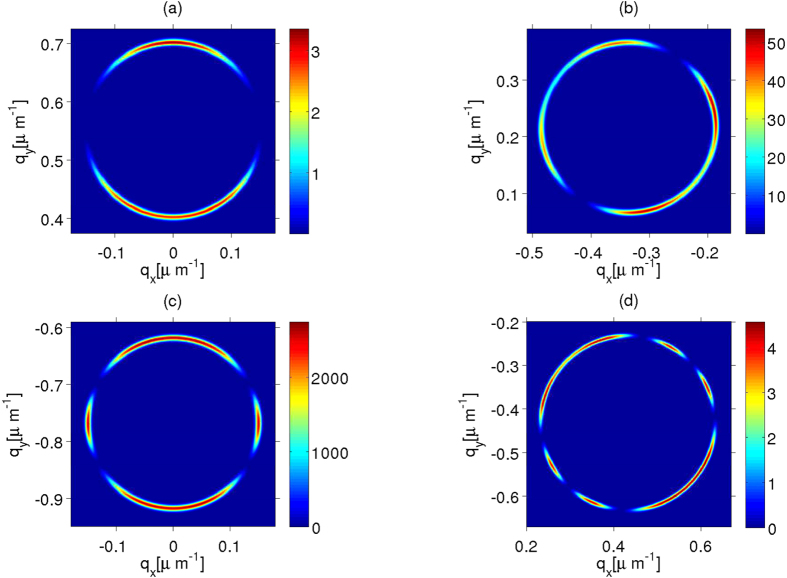
Flux rate of idler photons detections in coincidence with a signal photon with the transverse wavevector **p** that maximizes *R*_*s*_; the maximal **p** values depend on the point symmetry group for the axicon angle of the pump beam under consideration. The center of each resulting ring in **q** space is approximately −**p**, that is, the negative of the transverse wave vector of the companion signal photon. We consider type I degenerate SPDC in a nonlinear crystal of length *L* = 1 mm: (**a**) GaSe; (**b**) BBO; (**c**) LiNbO_3_ and (**d**) KDP. The incident beam is a Bessel TM mode with wavelength *λ* = 407 nm, 
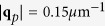
 for (**a**–**c**) and 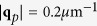 for (**d**). As in Fig. 2, we consider a spread of values of **q**_*p*_ of Δ**q**_*p*_ = 1/150 *μ*m, and the reported values correspond to the expected photon counts per second that would be obtained with a resolution detection of 0.005 *μ*m^−1^ and a pump beam of 30 mW.

The coincidence detections, given by Eq. (10) provide complementary useful information about the symmetry group of the medium under investigation. A direct calculation shows^22^ that for 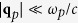, the detection of a signal photon with transverse wavevector **p** is accompanied by the detection of an idler photon that is confined to a ring with radius 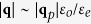 around **q** = −**p**. As **q**_*p*_ increases, the symmetry of the crystal becomes more visible in the angular distribution of idler photons, the flux rate becomes inhomogeneous along the ring with a structure that depends on the symmetry of the crystal, and which is compatible with the conditional generation of stationary Bessel photons, i. e. photons with an angular spectra resulting from superpositions of *m* and −*m* Bessel modes. The conservation of optical angular momentum restricts the possible values of *m*_*i*_ and *m*_*s*_ depending on which components of the nonlinear tensor 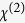 are different from zero^22^, a property that is directly related to the point symmetry group of the crystal. In Fig. 3, this effect is illustrated for various types of crystals. In the GaSe case (Fig. 3(a)), the conditional angular spectrum is a superposition of *m*_*i*_ = ±1 Bessel modes. This agrees with the option *m*_*s*_ = 0 and *m*_*i*_ = ±1, expected^22^ from the fact that for GaSe the dominant component of the nonlinear tensor is 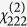
21. For the BBO crystal a dominant component of the nonlinear tensor is again 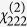, and also 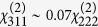; the resulting maximal conditional spectrum shown in Fig. 3(b) is a superposition of *m*_*i*_ = ±1 and *m*_*i*_ = ±3 with a small contributions of *m*_*i*_ = 0 which is also congruent with *m*_*s*_ = 0 from the expectations derived from ref. 22. For LiNbO_3_ 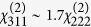 and the conditional angular spectrum, Fig. 3(c), is the superposition of *m*_*i*_ = 0, ±2, ±4, ±6 with a dominant contribution of *m*_*i*_ = ±2 that again is congruent with a dominant emission of *m*_*s*_ = 0 signal photons. The last case illustrated in Fig. 3(d) corresponds to KDP for which 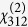 is a dominant component; the Fourier series of the maximal conditional angular spectrum in the *ϕ*_**q**_ variable requires also several values of *m*_*i*_, however the dominant ones yield a companion signal photon described by a Fourier series with several non negligible terms with *m*_*s*_ ≠ 0 values. Note that these results imply that the selection of the adequate point symmetry of a nonlinear crystal can enhance the generation of individual Bessel photons with predesigned angular momentum content^23^,^24^.

## Discussion

Summarizing, we have shown that, under the adequate experimental set up, the correlations of the photons emitted in a SPDC process, contain crystallographic information that can be accessed by measurements of the SPDC angular spectra and the conditional angular spectra. It is expected that a complete characterization of the twin photons correlations as a function of the properties of the structured pump beam could be used to obtain precise measurements of the first and second order electric susceptibility tensors. The results shown in this manuscript refer to the particular case of a beam impinging into an uniaxial crystal with the optic axis parallel to the normal of the crystal surface, other orientations of the relevant axes deserve further analysis. Preliminary numerical results show that the angular spectra loses gradually the direct visualization traces of the symmetry group as the orientation of the optic axis departs from the normal of the crystal surface. This makes the identification of the symmetry group a more computational demanding issue, that would be always based in the comparison between the observed angular spectra and the expected spectra from numerical simulations. Such a comparison would yield information not just on the symmetry group but also on the specific values of the electric susceptibility tensors. A similar treatment could be used for biaxial crystals.

It is also important to mention that the general procedure described in this work has facets that require a quantum analysis but also shares many features with its classical analogs. Phase matching conditions have clearly a classical interpretation; meanwhile, the conditional angular spectrum is by definition a quantum observable.

In general SPDC is a weak effect as noticed from the flux rates expected from moderate power of the pump beam. Recent advances in weak signal detection, e.g. the so called intensified cameras, make feasible the measurement of the flux rates shown in last section. Nevertheless, the effective pump power can be greatly enhanced using pulsed Bessel pump beams^25^ yielding, as a consequence, great enhancement of the down converted photons flux rates. For the theoretical study of SPDC with ultrashort pump pulses, the formalism described in this work can be extended to incorporate effects of the spectral density *α*(*ω*_*P*_) of the pump beam. Basically the coupling factor *g*, Eq. (8), becomes a frequency dependent term *α*(*ω*_*P*_)*g* that must be integrated over the involved frequency range. That kind of approach to SPDC has been studied experimentally in the context of Gaussian beams for which other interesting effects have been reported^26^.

Notice that the benefits arising from structured pump beams for nonlinear crystallography should not be restricted to the parametric down conversion process. Second harmonic generation is the most widely used nonlinear mechanism for crystallographic analyzes^7^,^8^,^27^. In fact, the adequate selection of the electromagnetic waves involved in SHG surmounts the intrinsic limitation of any nonlinear electromagnetic process induced by the bulk electric susceptibility 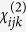: the necessity of dealing with noncentrosymmetric materials so that inversion symmetry is guaranteed. The key point is to induce combined electric dipole and magnetic dipole transitions^28^,^29^. Enhancement of this otherwise rare effect requires both the proper selection of the frequencies involved, so that the nonlinear process is resonant in both the incoming and outgoing light waves, and the usage of pulsed pump beams to achieve high intensities (typically pulses of tens of mJ in a pair of nanoseconds). External magnetic fields can also be used to manipulate the ferromagnetic domains. An outcome of such studies is the characterization of the magnetic point group of a material^27^. Nonlinear harmonic generation crystallography with pulsed Bessel beams could be an alternative to rotational anisotropy measurements, since using pulsed Bessel modes as a pump is equivalent to simultaneous measurements along different directions in a single shot. This idea deserves further studies which should have in mind tailoring of all the properties at hand of the conical superposition of plane waves depending on the susceptibility to be measured. They include: its spectral content *α*(*ω*), its angular spectra *β*(*ϕ*_**q**_) (taken here as 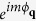, Eq. (2)), and its polarization.

## Additional Information

**How to cite this article**: Jáuregui, R. and Torres, J. P. On the use of structured light in nonlinear optics studies of the symmetry group of a crystal. *Sci. Rep.* **6**, 20906; doi: 10.1038/srep20906 (2016).
